# Corrigendum: The effect of brown seaweed and polyphenol supplementation in male rabbits on the blood profile and antioxidant markers

**DOI:** 10.17221/4/2023-VETMED

**Published:** 2023-01-18

**Authors:** Martin Massanyi

**Affiliations:** Department of Animal Husbandry, Faculty of Agrobiology and Food Resources, Slovak University of Agriculture, Nitra, Slovak Republic

Corrected were the sources of funding, page: 527.

The authors apologise for any inconvenience that it may have caused.

Original title page data:

“Supported by VEGA 1/0539/18: VEGA 1/0392/20, APVV-16-0289; Operational program – Integrated Infrastructure within the project: Demand-driven research for the sustainable and innovative food, Drive4SIFood 313011V336; co-financed by the European Regional Development Fund.”

Corrected title page data:

“Supported by the Slovak Research and Development Agency under the contracts no. APVV-16-0289 and no. APVV-21-0168. This publication was also supported by VEGA 1/0392/20, VEGA 1/0698/22 and by the Operational program - Integrated Infrastructure within the project: Demand-driven research for the sustainable and innovative food, Drive4SIFood 313011V336, co-financed by the European Regional Development Fund.”

